# Deletion of the B-B’ and C-C’ regions of inverted terminal repeats reduces rAAV productivity but increases transgene expression

**DOI:** 10.1038/s41598-017-04054-4

**Published:** 2017-07-14

**Authors:** Qingzhang Zhou, Wenhong Tian, Chunguo Liu, Zhonghui Lian, Xiaoyan Dong, Xiaobing Wu

**Affiliations:** 1 College of Life Sciences, Jilin University, Changchun, Jilin China; 2 Beijing Ruicy Gene Therapy Institute for Rare Diseases, Beijing, China; 3 College of Life Science and Technology, Beijing University of Chemical Technology, Beijing, China; 4 Beijing FivePlus Molecular Medicine Institute, Beijing, China

## Abstract

Inverted terminal repeats (ITRs) of the adeno-associated virus (AAV) are essential for rescue, replication, packaging, and integration of the viral genome. While ITR mutations have been identified in previous reports, we designed a new truncated ITR lacking the B-B’ and C-C’ regions named as ITRΔBC and investigated its effects on viral genome replication, packaging, and expression of recombinant AAV (rAAV). The packaging ability was compared between ITRΔBC rAAV and wild-type (wt) ITR rAAV. Our results showed the productivity of ITRΔBC rAAV was reduced 4-fold, which is consistent with the 8-fold decrease in the replication of viral genomic DNA of ITRΔBC rAAV compared with wt ITR rAAV. Surprisingly, transgene expression was significantly higher for ITRΔBC rAAV. A preliminary exploration of the underlying mechanisms was carried out by inhibiting and degrading the ataxia telangiectasia mutated (ATM) protein and the Mre11 complex (MRN), respectively, since the rAAV expression was inhibited by the ATM and/or MRN through *cis* interaction or binding with wt ITRs. We demonstrated that the inhibitory effects were weakened on ITRΔBC rAAV expression. This study suggests deletion in ITR can affect the transgene expression of AAV, which provides a new way to improve the AAV expression through ITRs modification.

## Introduction

Adeno-associated virus (AAV) is a nonpathogenic member of the *Parvoviridae* family^1^ that has received much attention as a promising vector for gene therapy and has been assessed in clinical trials^2^. The wild-type (wt) AAV genome contains inverted terminal repeats (ITRs) that consist of 145 nucleotides (nt) at both ends. The terminal 125 nt of each ITR self-anneals to form a palindromic double-stranded T-shaped hairpin (HP) structure, in which the small palindromic B-B’ and C-C’ regions form the cross arm and the large palindromic A-A’ region forms the stem. Each HP is followed by a unique 20-nt D (or D’) region^1, 3^. Although ITRs are usually 145 nt in length, recombinant AAV (rAAV) production is not affected by wt ITRs of 137 nt or less, which lack several nucleotides including the 5′ terminal resolution site (trs). Because the 137-nt ITRs are restored to 145 nt during genome replication^4^. The presence of ITRs is the sole *cis* requirement for rescue, replication, packaging, and integration of AAV genomes^4–9^.

ITRs are very important for AAV, but various deletions have been identified in rAAV vector plasmids. We found the B-B’ region in the upstream ITRs was lost in the pAAV2neo, which was a rAAV vector plasmid in our laboratory, although this deletion did not impact the packaging function of viral vector. Samulski *et al*.^4^ reported that deletion of 113 base pairs (bp) in one ITR or deletion of 11 bp in the C-C’ region of one ITR can be corrected. Moreover, Wang *et al*.^10^ found that AAV genomes can be rescued, replicated, and successfully packaged despite the absence of one HP structure. The diversity of ITR deletions has suggested that some ITR regions maybe dispensable for AAV packaging. In addition, Chiorini *et al*.^11^ reported that the ability of Rep68 to bind to ITRs does not require the presence of the hairpin cross arm (the B-B’ and C-C’ regions). The ability of Rep68 to bind wt hairpin ITRs or mutant ITRs in which the B-B’ and C-C’ regions were deleted was identical. However, given the importance of ITRs to AAV, mutant ITRs are rarely used in rAAV packaging. And as a result, studies on the effects of mutant ITRs on AAV have been limited. It is unclear whether removal of the B-B’ and C-C’ regions from ITRs impairs the packaging and transgene expression of rAAV. To address these issues, we designed a rAAV vector plasmid harboring two mutant ITRs that lacked the B-B’ and C-C’ regions, and examined the effects of ITR truncation on packaging and expression of rAAV.

## Results

### Construction of a rAAV vector plasmid with two truncated ITRs lacking the B-B’ and C-C’ regions

**Figure 1:**
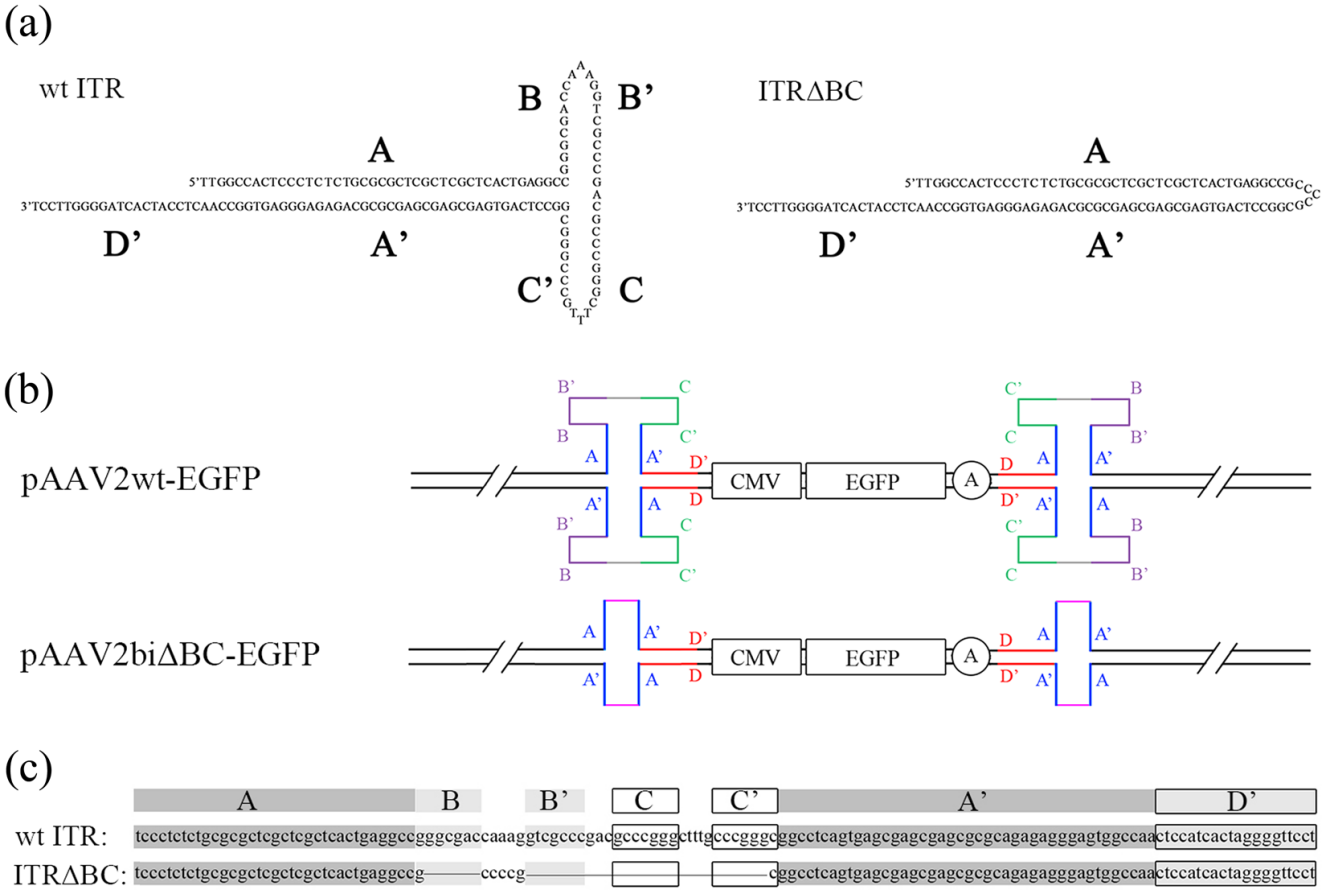
Construction of the rAAV vector with deletion of the B-B’ and C-C’ regions in the two ITRs. (**a**) Nucleotide sequence and schematic diagram of T-shaped wt ITR and U-shaped ITRΔBC. The wt ITR contains three palindromic sequences (A-A’, B-B’, and C-C’ regions) and forms a T-shaped structure, while ITRΔBC includes only one palindromic sequence (A-A’ region) and forms a U-shaped structure. (**b**) rAAV vector plasmids. The conventional rAAVwt-EGFP vector plasmid pAAV2wt-EGFP has two wt ITRs flanking the CMV-EGPF-pA expression cassette, while the rAAVbiΔBC-EGFP vector plasmid pAAV2biΔBC-EGFP has two ITRΔBCs. (**c**) Sequencing of wt ITRs and ITRΔBCs in pAAV2wt-EGFP and pAAV2biΔBC-EGFP. Only one ITR in the upstream direction of the expression cassette is shown. The second ITR was identical. Primer synthesis and DNA sequencing were performed by Takara.

To determine whether the B-B’ and C-C’ regions in the ITRs are dispensable for rAAV packaging, we designed and constructed a novel truncated ITR lacking the B-B’ and C-C’ regions (ITRΔBC). In ITRΔBC, the A and A’ regions are connected by five random nucleotides (5′-CCCCG-3′). Unlike wt ITRs, ITRΔBC forms a simple palindrome similar in shape to the letter U, which we designated as a U-shaped ITR (Fig. 1a). It was synthesized and used to replace the two wt ITRs in our rAAV vector plasmid pAAV2wt to generate the pAAV2biΔBC. An enhanced green fluorescence protein (EGFP) expression cassette with a cytomegalovirus (CMV) promoter was used as a reporter (Fig. 1b). The construction of pAAV2biΔBC-EGFP and pAAV2wt-EGFP was verified by enzyme digestion and sequencing. Our confirmation revealed that two wt ITRs were present in pAAV2wt-EGFP, and pAAV2biΔBC-EGFP contained two mutant ITRs lacking the B-B’ and C-C’ regions (Fig. 1c; Supplementary Fig. S1, Fig. S2).

### rAAV with two ITRΔBCs can be encapsulated but with lower productivity

**Figure 2:**
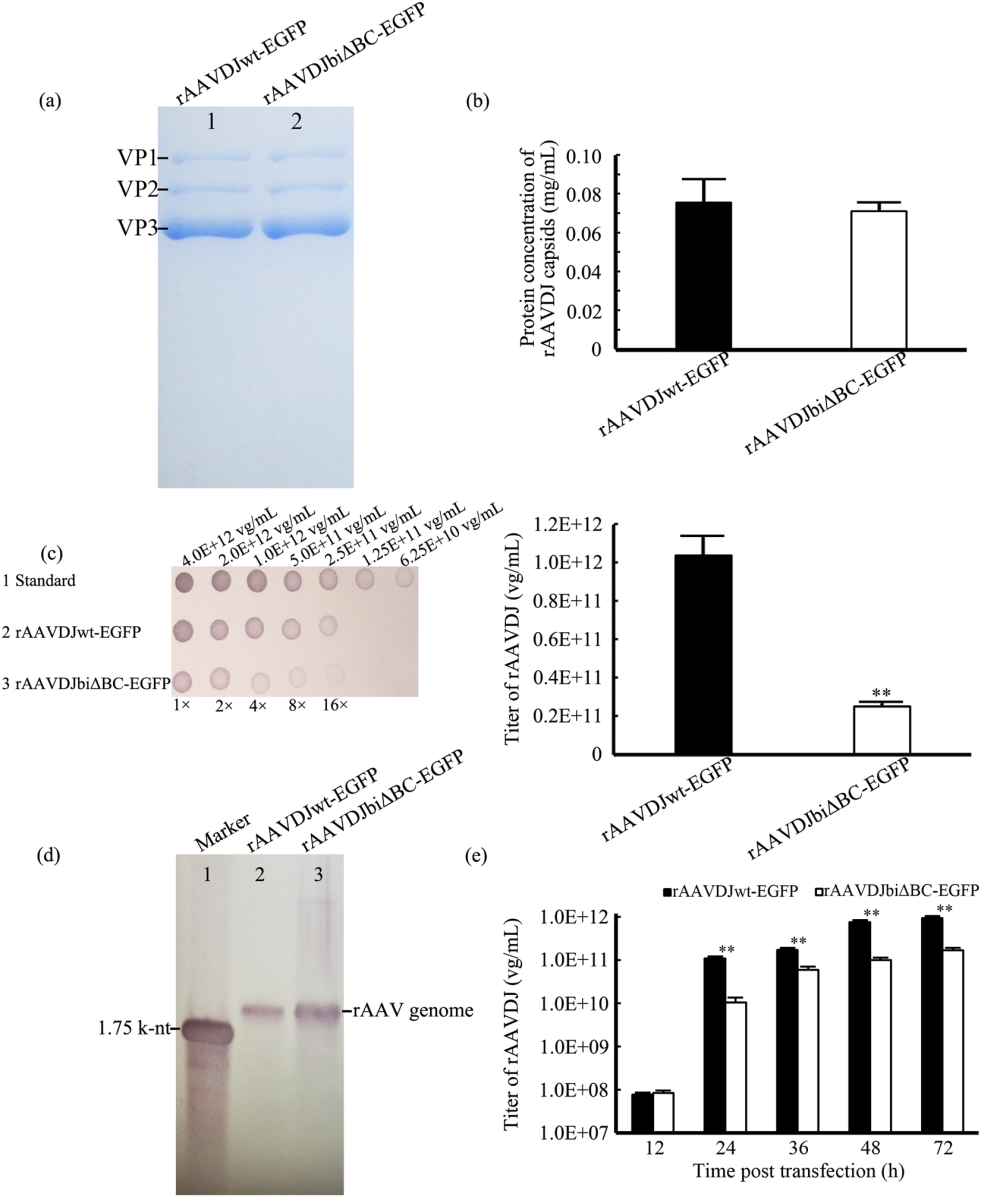
Effect of the deletion of the B-B’ and C-C’ regions in the two ITRs on rAAV productivity. (**a**) SDS-PAGE analysis of the viral capsids. There were no other proteins present except the viral capsids VP1, VP2, and VP3. (**b**) Concentrations of the capsid proteins of rAAVDJwt-EGFP and rAAVDJbiΔBC-EGFP. (**c**) The titers of rAAVDJwt-EGFP and rAAVDJbiΔBC-EGFP. Titers were detected by DNA dot blot (left) and qPCR (right). All types of rAAV were packaged five times simultaneously. Bars represent standard deviation (SD) of the mean (*n* = 5). (**d**) Viral genome integrity. Genomic integrity of rAAVDJwt-EGFP and rAAVDJbiΔBC-EGFP was determined by Southern blotting. SDS-PAGE, DNA dot blot, and Southern blotting results from the first batch of rAAV are shown. (**e**) Titers of rAAVDJwt-EGFP and rAAVDJbiΔBC-EGFP purified at different time points 72 h following rAAV vector plasmids transfection. Bars represent SD of the mean (*n* = 3). A Student’s paired *t*-test was used to assess the significance. Pairwise comparisons between groups, **p < 0.01. Pictures of SDS-PAGE (**a**) and Southern blotting (**d**) were cropped. The original full-length pictures were shown in Supplementary Fig. S4.

pAAV2wt-EGFP and pAAV2biΔBC-EGFP were used in rAAV vector packaging based on serotype DJ^12^, five times under identical conditions with the Adeno virus-free, triple-plasmid co-transfection method^13^. The two types of rAAV vectors were named rAAVDJwt-EGFP and rAAVDJbiΔBC-EGFP, respectively. We used multiple methods to determine the packaging efficiency. Initially, SDS-polyacrylamide gel electrophoresis (SDS-PAGE) analysis showed that only the viral capsid proteins VP1, VP2, and VP3 were present in purified rAAV (Fig. 2a), indicating that our purification method yielded high purity rAAV^14^. Using the bicinchoninic acid (BCA) assay, we determined the concentration of the viral capsid proteins and found no differences between rAAVDJwt-EGFP and rAAVDJbiΔBC-EGFP (Fig. 2b), indicating a similar number of particles in both. To further compare the productivity of these two rAAV vectors, we first adjusted the concentrations of the capsid proteins to be the same, and used DNA dot blot assays to determine the titers. The viral DNA genome was extracted and hybridized with probes derived from the CMV promoter. Since titers detected by the DNA dot blot method may show 50% variation, we also used quantitative PCR (qPCR) for more accurate detection. Titers were only accepted when they were shown to be consistent with both methods. The rAAV titers we showed in this study were determined by qPCR because of its greater precision. The titer of rAAVDJwt-EGFP was determined to be (1.03 ± 0.15) × 10^12^ viral genomes (vg)/mL, while rAAVDJbiΔBC-EGFP was (2.50 ± 0.23) × 10^11^ vg/mL, which is only about 25% of the former (Fig. 2c, Supplementary Tab. S1, Fig. S3). As an important indicator, genomic integrity was also assessed^15^. Southern blotting analysis of purified viral DNA from rAAVDJbiΔBC-EGFP and rAAVDJwt-EGFP indicated that viral genomic DNA from both vectors was approximately 2.0 kilo-nucleotide (k-nt). Similar to rAAVDJwt-EGFP, a single band corresponding to genomic DNA of single stranded rAAVDJbiΔBC-EGFP (ssAAV) was apparent, but a slight band of self-complementary rAAVDJbiΔBC-EGFP (scAAV) was also apparent, suggesting that the majority of the rAAVDJbiΔBC-EGFP genome was homogeneous and intact (Fig. 2d).

Deletion of the B-B’ and C-C’ regions of the ITRs did not affect rAAV genome encapsulation, but viral productivity was reduced by 75%. To further confirm these differences, rAAVDJwt-EGFP and rAAVDJbiΔBC-EGFP were purified at different time points during a 72-h timecourse following transfection of the rAAV vector plasmids with the Adeno virus-free, triple-plasmid co-transfection method. After adjusting the capsid protein concentration to be the same at each time point, the titers of rAAVDJwt-EGFP and rAAVDJbiΔBC-EGFP were assayed by DNA dot blot assay and qPCR. We found that only at 12 h post-transfection were the two titers similar. During the timecourse, the titer of rAAVDJwt-EGFP began to exceed that of rAAVDJbiΔBC-EGFP and the differences gradually intensified with time (Fig. 2e). The lower titer of rAAVbiΔBC at the different time points further confirmed that deletion of the B-B’ and C-C’ regions in the ITRs resulted in a decrease in rAAV productivity.

### Replication efficiency of the rAAV genome was decreased by deletion of the B-B’ and C-C’ regions in the ITRs

**Figure 3:**
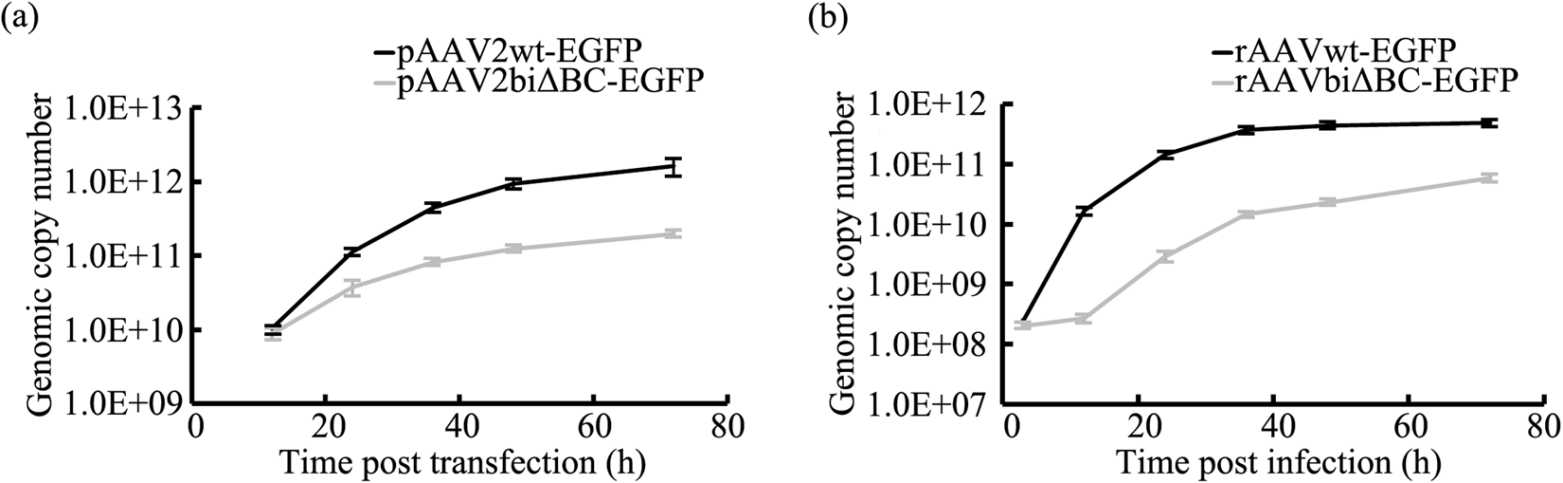
Replication efficiency is affected by deletion of the B-B’ and C-C’ regions in the two ITRs. (**a**) Replication of the rAAV genome following co-transfection with pHelper, pcDNA3.1-p5-Rep, and rAAV vectors plasmids in HEK293 cells. Hirt DNA was extracted at different time points post-transfection. (**b**) Replication of the rAAV genome following pHelper and pcDNA3.1-p5-Rep co-transfected HEK293 cells infected with rAAV. Hirt DNA was extracted at different time points post-transfection. Bars represent SD of the mean (*n* = 3).

The packaging efficiency of rAAV closely related to replication of viral genome. The low productivity of rAAVbiΔBC suggested that replication of the rAAV genome may be affected by deletion of the B-B’ and C-C’ regions in the two ITRs. To investigate the replication of the rAAVbiΔBC genome, human embryonic kidney cells 293 (HEK293) were co-transfected with pHelper, Rep expression and rAAV vector plasmids by calcium phosphate DNA precipitation. After expression of the Rep protein, the AAV genome was rescued from the plasmid with trs nicking and replicated^6, 16^. The viral genomic copy number was detected continuously from 0 to 72 h post-transfection in Hirt DNA. As shown in Fig. 3a, the copy number of rAAVwt-EGFP was greater than rAAVbiΔBC-EGFP at all time points except the initial 12 h post-transfection. Although the initial genomic copy number of the two types of rAAV was approximate, the growth of rAAVbiΔBC-EGFP was significantly lower than rAAVwt-EGFP over time. In the final time point at 72 h post-transfection, rAAVwt-EGFP and rAAVbiΔBC-EGFP were 154.25- and 22.13-fold higher, respectively, than at the 12-h time point. The rAAVbiΔBC-EGFP copy number was only 12.14% of rAAVwt-EGFP. Compared with the control, the low copy number of rAAVbiΔBC-EGFP suggested that deletion of the B-B’ and C-C’ regions in the ITRs results in a significant decrease in the replication efficiency of the rAAV genome.

To further compare the replication efficiency of the rAAVbiΔBC and rAAVwt genomes, we used direct rAAV infection instead of transfection of the rAAV vector plasmid, eliminating any interference introduced by the rescue of viral genomes. After HEK293 cells were co-transfected for 12 h with pHelper and Rep expression plasmids, the HEK293 cells were infected with rAAVDJwt-EGFP and rAAVDJbiΔBC-EGFP at a multiplicity of infection (MOI) of 200 vg/cell. In the following 72 h, the viral copy number in Hirt DNA was assayed with qPCR. Prior to infection, the titers of the two types of rAAV were adjusted to be the same (Supplementary Fig. S5). The copy numbers of rAAVbiΔBC-EGFP and its control were similar 3 h post-infection, indicating the initial replication numbers were similar. However, rAAVwt-EGFP began to exceed rAAVbiΔBC-EGFP and distinct differences were apparent throughout the timecourse. From 3 to 72 h post-infection, the copy number of rAAVwt-EGFP increased 2030-fold, while rAAVbiΔBC-EGFP only increased 266-fold. And the copy number of rAAVwt-EGFP was 8.28-fold that of rAAVbiΔBC-EGFP 72 h post-infection (Fig. 3b). These differences also suggest that deletion of the B-B’ and C-C’ regions in the ITRs impairs the rAAV replication efficiency.

### rAAV with two ITRΔBCs is capable of efficient transgene expression *in vitro* and *in vivo*

**Figure 4:**
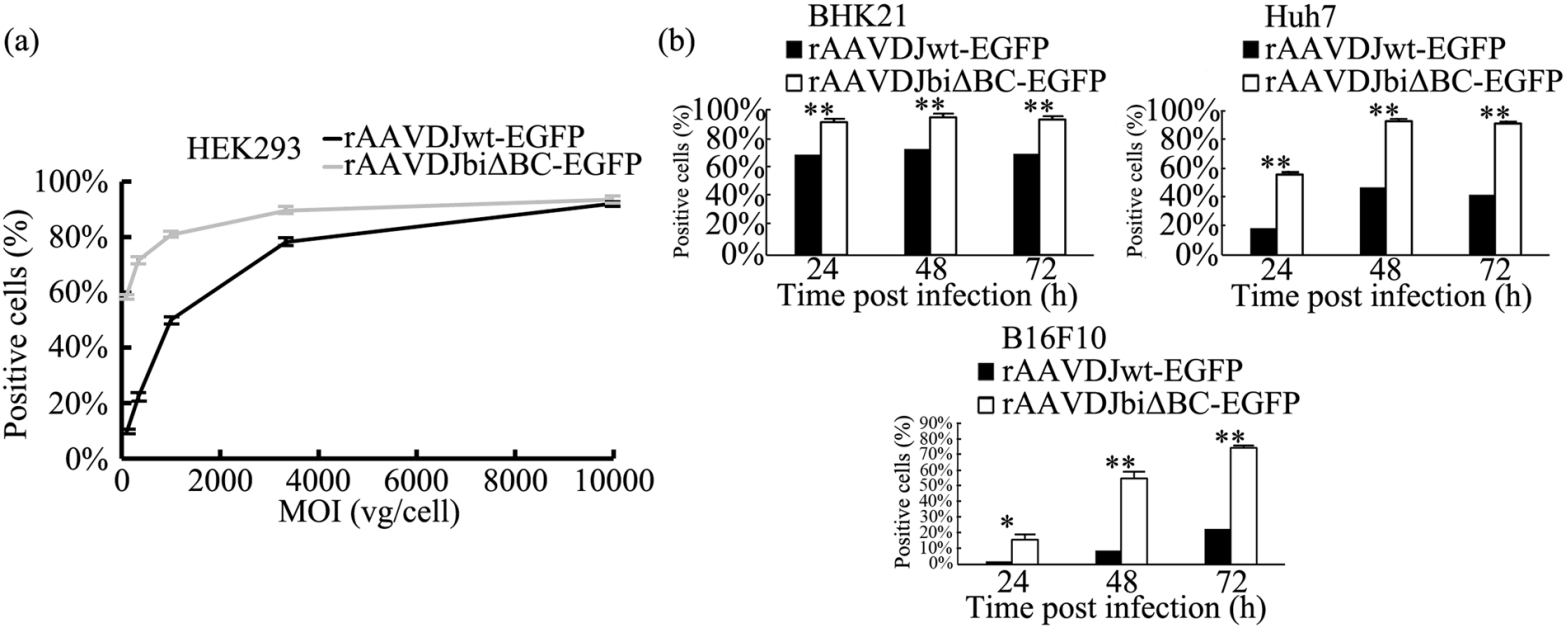
*In vitro* expression of rAAV. (**a**) Transgene expression of rAAVDJwt-EGFP and rAAVDJbiΔBC-EGFP at a series of MOIs ranging from 100 to 10,000 vg/cell in HEK293 cells. EGFP expression was quantified by flow cytometry, and is shown as the percentage of cells expressing EGFP. The percentage of positive cells infected with rAAVDJbiΔBC-EGFP was significantly higher than rAAVDJwt-EGFP at all the MOIs tested except 10,000 vg/cell. (**b**) Transgene expression of rAAVDJwt-EGFP and rAAVDJbiΔBC-EGFP at a dose of 1,000 vg/cell in BHK21, Huh7, and B16F10 cells. The expression of rAAVDJbiΔBC-EGFP was significantly higher than rAAVDJwt-EGFP at all time points. Bars represent SD of the mean (*n* = 3). A Student’s paired *t*-test was used to assess the significance. Pairwise comparisons between groups, *p < 0.05 and **p < 0.01.

It has been shown that, compared with rAAVwt, productivity of rAAVbiΔBC is reduced as a result of the decrease in replication. It is unclear, however, whether the deletion in the ITRs is harmful to transgene expression. Therefore, we compared transgene expression of rAAVbiΔBC and rAAVwt *in vitro*. To avoid interference by quantitative error, HEK293 cells were infected with rAAVDJwt-EGFP and rAAVDJbiΔBC-EGFP at a series of MOIs ranging from 100 to 10,000 vg/cell and EGFP expression was detected 48 h post-infection. The percentage of green fluorescent HEK293 cells infected with rAAVDJbiΔBC-EGFP was 58.8–93.4%, while the percentage of cells infected with rAAVDJwt-EGPF ranged from 9.54 to 92.2%. The proportion of cells infected with rAAVDJbiΔBC-EGFP was only similar to rAAVDJwt-EGFP at an MOI of 10,000 vg/cell. As the MOI decreased, the numbers of cells infected with rAAVDJbiΔBC-EGFP were higher than the control (Fig. 4a). In baby hamster kidney fibroblasts 21 (BHK21) and Huh7 cells, two cell lines highly permissive to AAV, EGFP expression was detected at 24, 48, and 72 h post-infection at an MOI of 1,000 vg/cell. rAAVDJbiΔBC-EGFP infection resulted in more EGFP-positive cells at all time points. We then examined B16F10 cells, which have low permissivity to rAAV. Although the proportions were lower, the same trend was apparent as with the other cell lines tested (Fig. 4b, Supplementary Fig. S6). Transgene expression of rAAVbiΔBC was higher than rAAVwt in all tests, which indicated that deletion of the B-B’ and C-C’ regions in the two ITRs increased the transgene expression of rAAV *in vitro*.

**Figure 5:**
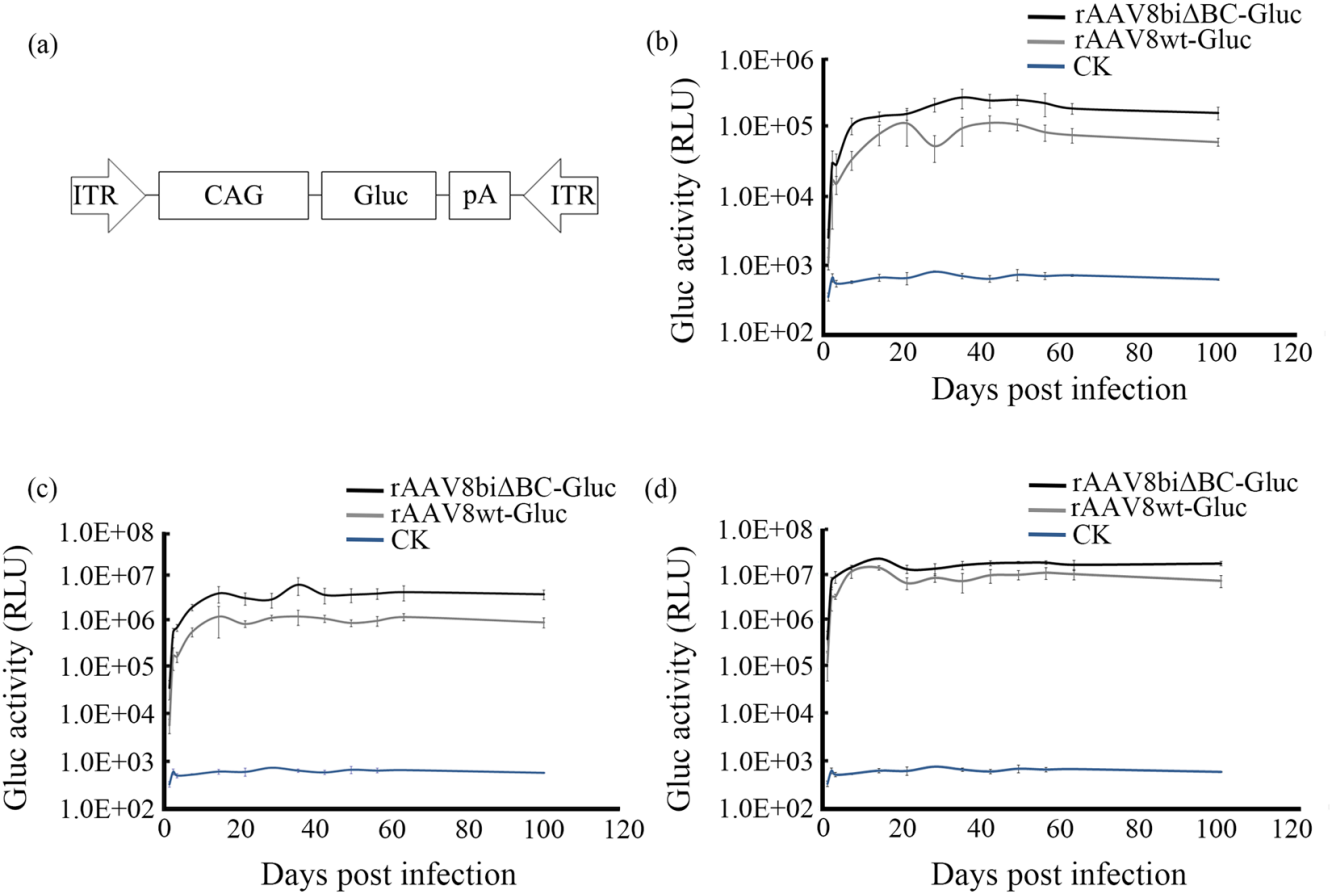
*In vivo* expression of rAAV. Deletion of the B-B’ and C-C’ regions in the ITRs increased transgene expression of rAAV. (**a**) Schematic of the rAAV8-Gluc genome used to infect C57BL/6 J mice. Expression of rAAV8wt-Gluc and rAAV8biΔBC-Gluc following tail vein injection into C57BL/6 J mice at doses of 2 × 10^9^ vg (**b**), 2 × 10^10^ vg (**c**), and 2 × 10^11^ vg (**d**). Gluc activity in the blood was assayed 1–100 d post-injection. The CK group of mice was injected with PBS. Bars represent SD of the mean (*n* = 5).

Since *in vivo* expression of rAAV is more widely used, we also determined whether deletion of the B-B’ and C-C’ regions in the two ITRs could increase rAAV expression *in vivo*. To accurately detect the expression in individual animals over time, *Gaussia* luciferase (Gluc), a secreted luciferase with high sensitivity and with expression driven by the CAG promoter, was used as a reporter^17–19^. We generated rAAV8-Gluc vectors with wt ITRs and ITRΔBCs, respectively (Fig. 5a). After the titers were adjusted, C57BL/6 J mice were injected via the tail vein with rAAV8wt-Gluc and rAAV8biΔBC-Gluc at doses of 2.0 × 10^11^ vg (high dose), 2.0 × 10^10^ vg (intermediate dose), and 2.0 × 10^9^ vg (low dose). The Gluc activity of the tail vein blood was monitored continuously 1–100 d post-injection. Our results indicated that Gluc expression in mice injected with rAAV8biΔBC-Gluc was higher at all time points while only minimum activity was detected in mice injected with rAAV8wt-Gluc. In the intermediate dose group, rAAV8biΔBC-Gluc exhibited 2.51–6.66-fold higher Gluc activity. In the other two groups, the Gluc activity of mice injected with rAAV8biΔBC was 1.38–3.13- and 1.24–3.21-fold higher than the respective controls in the high- and low-dose groups, respectively (Fig. 5b–d), indicating that rAAVbiΔBC can be effectively expressed *in vivo*. Expression efficiency of rAAVbiΔBC was also higher than rAAVwt, consistent with *in vitro* expression. Moreover, from 14 to 100 d post-infection, the Gluc activity of rAAV8biΔBC in the three dosage groups remained stable, suggesting that the deletion in the ITRs did not reduce the persistence of rAAV expression for at least three months *in vivo*.

### rAAVbiΔBC can accommodate large genome (about 4.4 k-nt) with highly efficient expression

**Figure 6:**
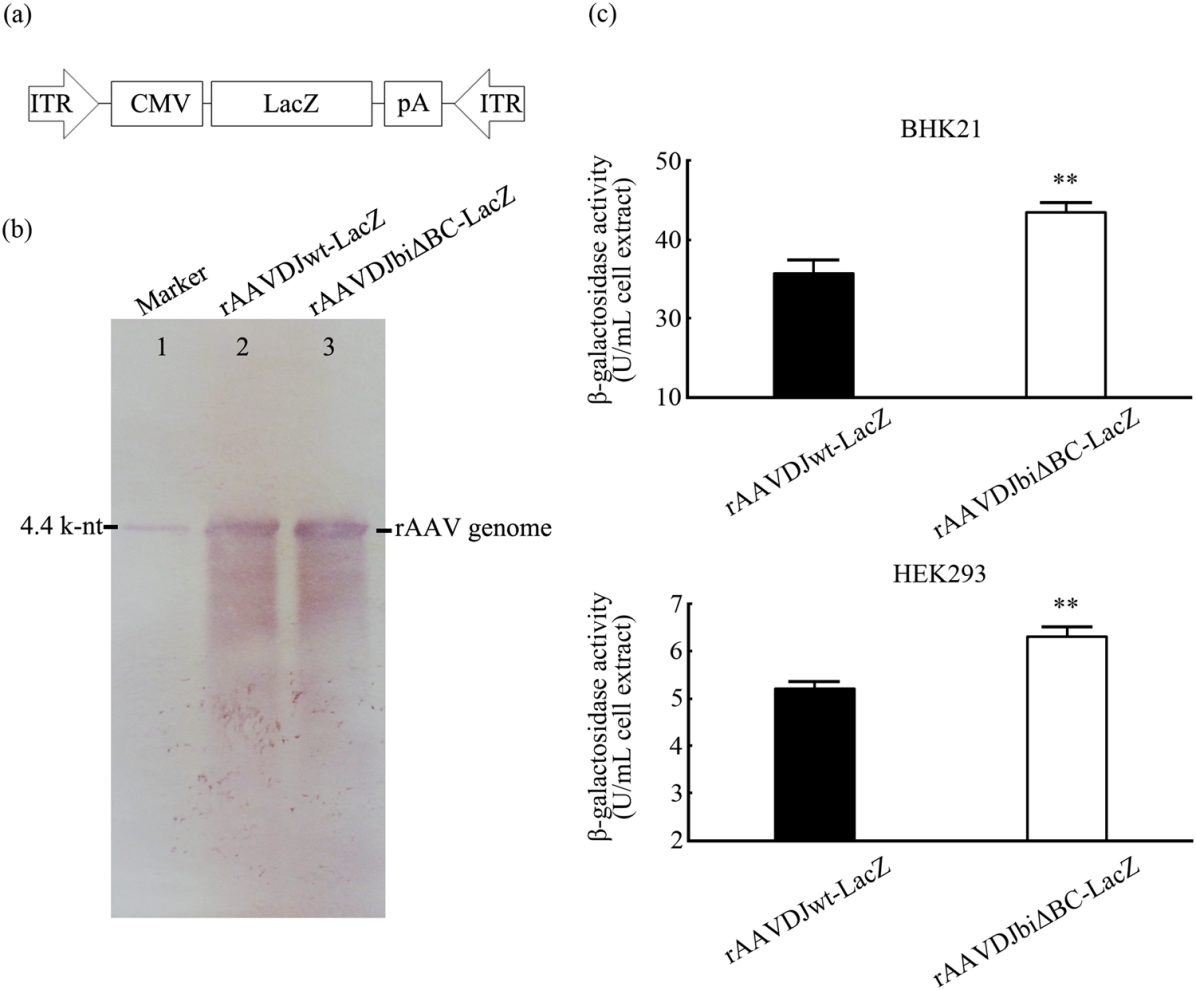
High expression of rAAVDJbiΔBC-LacZ. (**a**) Schematic of the rAAVDJ-LacZ genome. (**b**) Genomic integrities of rAAVDJwt-LacZ and rAAVDJbiΔBC-LacZ were determined by Southern blotting. (**c**) β-galactosidase activity. BHK21 and HEK293 cells were infected with rAAVDJwt-LacZ and rAAVDJbiΔBC-LacZ at an MOI of 10,000 vg/cell, 48 h post-infection, the β-galactosidase activity was determined. The expression of rAAVDJbiΔBC-LacZ was significantly higher than that of rAAVDJwt-LacZ. Bars represent SD of the mean (*n* = 3). A Student’s paired *t*-test was used to assess the significance. Pairwise comparisons between groups, **p < 0.01. Picture of Southern blotting (**b**) was cropped. The original full-length picture was shown in Supplementary Fig. S7.

Because the genome of rAAVDJbiΔBC-EGFP is approximately 2.0 k-nt, a small portion of scAAV is packaged. Whether the high expression of rAAVbiΔBC was because of the presence of scAAV was evaluated. To eliminate interference by scAAV, we packaged rAAVDJbiΔBC-LacZ and its control rAAVDJwt-LacZ. Both of them accommodated large genomes (about 4.4 k-nt) (Fig. 6a). DNA dot blot and qPCR assays demonstrated that the rAAVDJwt-LacZ titer was 4-fold that of rAAVDJbiΔBC-LacZ. Southern blotting indicated that viral genomic DNA from rAAVDJbiΔBC-LacZ and rAAVDJwt-LacZ was 4.4 k-nt in size. Bands corresponding to genomic DNA of single-stranded rAAVDJbiΔBC-LacZ and rAAVDJwt-LacZ (ssAAV) were apparent. No scAAV band was detected (Fig. 6b).

To assess the expression *in vitro*, BHK21 and HEK293 cells were infected with rAAVDJbiΔBC-LacZ and rAAVDJwt-LacZ at an MOI of 10,000 vg/cell. The β-galactosidase activity was determined 48 h post-infection. Although the activities differed between the two cell lines, that of rAAVDJbiΔBC-LacZ was higher than that of rAAVDJwt-LacZ in both of cell lines (Fig. 6c). This result suggested that high expression of rAAVbiΔBC may not be attributable to the presence of scAAV.

### Inhibition of ataxia telangiectasia mutated (ATM) function leads to a more increased in expression of rAAV with wt ITRs

**Figure 7:**
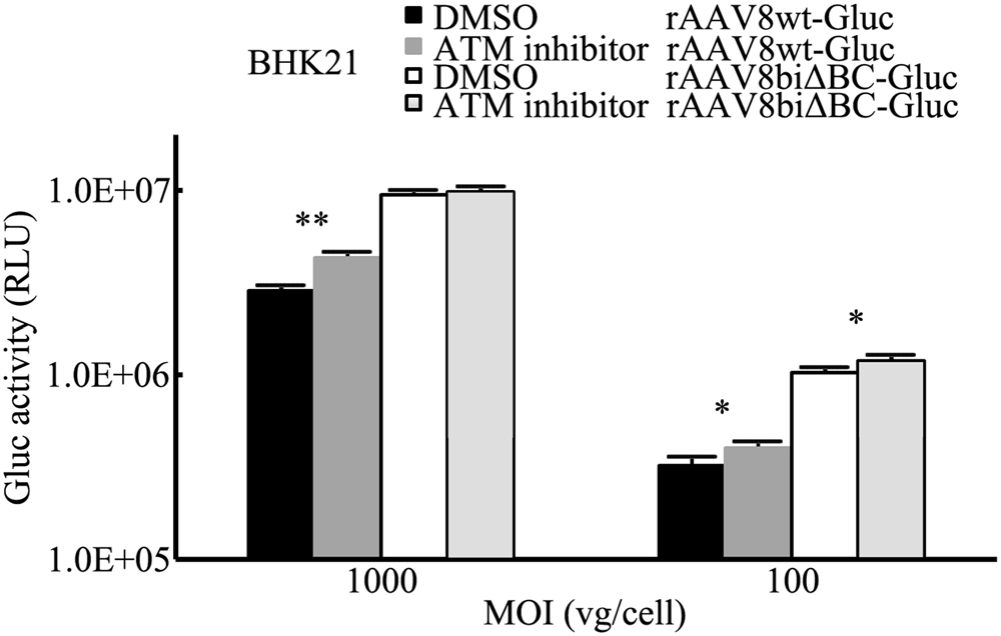
Effect of ATM on rAAV expression. BHK21 cells were infected with rAAV8wt-Gluc and rAAV8biΔBC-Gluc in the presence of 10 μM ATM inhibitor in DMSO or DMSO only. Gluc activity was assayed 48 h post-infection. The increase in the expression of rAAV8wt-Gluc was greater than rAAV8biΔBC-Gluc. Bars represent SD of the mean (*n* = 3). A Student’s paired *t*-test was used to assess the significance. Pairwise comparisons between groups, *p < 0.05 and **p < 0.01.

It has been reported that loss of function of the ATM protein enhances rAAV transduction^20^. Furthermore, studies have indicated that DNA molecules with a palindromic terminal repeat sequence constrained in a T-shaped hairpin conformation at one or both ends are subject to a loss of gene expression^21^. This loss of expression is caused by transcriptional silencing of T-shaped hairpin-containing molecules, and is mediated by ATM. However, DNA molecules with simple U-shaped hairpin ends are unaffected^21, 22^. Consequently, we assumed that ATM may affect the expression of rAAV with T-shaped wt ITRs, but rAAV with U-shaped ITRΔBCs would be unaffected. To confirm the assumption, BHK21 cells were infected with rAAV8wt-Gluc and rAAV8biΔBC-Gluc at a series of MOIs in the presence or absence of ATM inhibitor. Expression of rAAV8biΔBC-Gluc was only slightly affected by the ATM inhibitor 48 h post-infection. Particularly at an MOI of 1,000 vg/cell, Gluc activity only increased by 3.3% while rAAV8wt-Gluc increased by 50.7%. At an MOI of 100 vg/cell, the presence of the ATM inhibitor increased the Gluc activity of rAAV8wt-Gluc by 25.0%, a 1.6-fold increase compared with rAAV8biΔBC-Gluc (Fig. 7). Although inhibition of ATM also mildly enhanced the expression of rAAV8biΔBC-Gluc, the increase was far less than the control, suggesting that compared with U-shaped ITRΔBCs, T-shaped wt ITRs are more susceptible to ATM-dependent loss of AAV gene expression. This is likely one of the reasons for the increased expression of rAAV with ITRΔBCs.

### Reduction in the amount of Mre11/Rad50/Nbs1 complex results in a greater increase in expression of rAAV with wt ITRs

**Figure 8:**
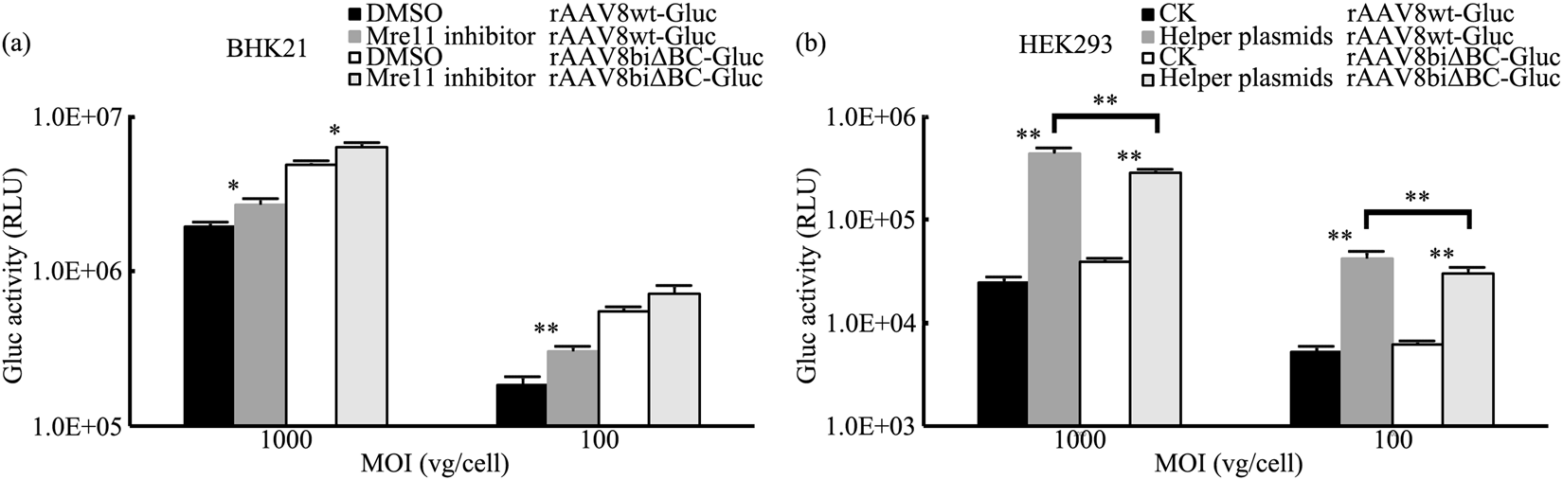
Effect of MRN on rAAV expression. (**a**) BHK21 cells were infected with rAAV8wt-Gluc and rAAV8biΔBC-Gluc in the presence of 25 μM Mre11 inhibitor in DMSO or DMSO only. Gluc activity was assayed 48 h post-infection. (**b**) In the experimental group rAAV8wt-Gluc and rAAV8biΔBC-Gluc infected HEK293 cells, which were transfected with pHelper plasmid 12 h prior; while in the control group HEK293 cells were directly infected with rAAV8wt-Gluc and rAAV8biΔBC-Gluc. Gluc activity was assayed 48 h post-infection. The increase in the expression of rAAV8wt was greater than rAAV8biΔBC. Bars represent SD of the mean (*n* = 3). A Student’s paired *t*-test was used to assess the significance. Pairwise comparisons between groups, *p < 0.05 and **p < 0.01.

The Mre11 complex (MRN), composed of Mre11, Rad50, and Nbs1, which is critical for DNA damage sensing, signaling, and repair, can limit AAV transduction. Degradation of MRN by the Ad E1b55K/E4orf6 protein complex enhances AAV transduction dramatically^23, 24^. Previous researches have shown this inhibition on rAAV expression is dependent on the recognition and interaction of MRN with ITRs in the AAV genome^23, 24^. Accordingly, we further determined whether MRN could inhibit the expression of rAAV with ITRΔBCs. BHK21 cells were infected with rAAV8wt-Gluc and rAAV8biΔBC-Gluc at a series of MOIs in the presence or absence of an Mre11 inhibitor. Although the Gluc activity of rAAV8biΔBC-Gluc was also increased in the presence of the Mre11 inhibitor, the increase was always less than that observed with rAAV8wt-Gluc 48 h post-infection. Especially at an MOI of 100 vg/cell, the increase in expression of rAAV8wt-Gluc was 63.98%, a 2.13-fold increase compared with rAAV8biΔBC-Gluc (30.10%). And at an MOI of 1000 vg/cell, the increase of rAAV8wt-Gluc was 38.69%, still higher than that (29.82%) of rAAV8biΔBC-Gluc (Fig. 8a). These results suggested that the MRN-dependent inhibitory effect on expression was more pronounced in rAAV with wt ITRs compared with ITRΔBCs. This may also explain the increased expression seen with rAAVbiΔBC.

To further examine whether the deletion of the B-B’ and C-C’ regions in the two ITRs helped to weaken MRN-dependent inhibitory effect on AAV expression, a MRN degradation experiment was done based on previous works^23–25^. HEK293 cells were infected with rAAV8wt-Gluc and rAAV8biΔBC-Gluc in presence or absence of transfection with the pHelper plasmid harboring the adenovirus helper E2, E4, and VA RNA genes. In presence of the pHelper plasmid transfection, both of the expressions of rAAV8wt-Gluc and rAAV8biΔBC-Gluc were increased, which could be explained by the MRN degradation. Furthermore, the increase of rAAV8biΔBC-Gluc expression was much less than its control (Fig. 8b). These results suggested that the inhibitory effect of MRN on rAAV expression mediated by wt ITR was stronger than ITRΔBC.

### Deletion of the B-B’ and C-C’ regions in the ITRs did not increase the expression of rAAV DNA as plasmid or genomic DNA extracted from virions

**Figure 9:**
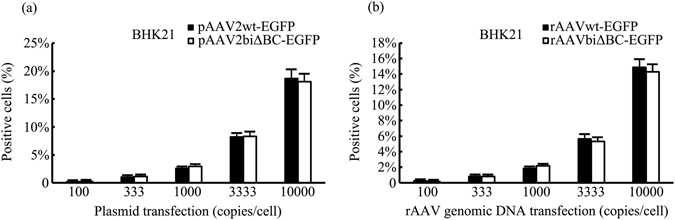
Expression of rAAVwt and rAAVbiΔBC introduced into cells as plasmids or genomic DNA extracted from virions. Expression of rAAV DNA molecules as plasmid DNA (**a**) or genomic DNA extracted from virions (**b**). Bars represent SD of the mean (*n* = 3).

We found that expressions with rAAVbiΔBC infection were always higher than rAAVwt. In order to determine whether these two types of viral vectors DNAs transduced cells as other forms could also lead to a difference in expression, rAAV vectors plasmid DNAs pAAV2wt-EGFP, pAAV2biΔBC-EGFP and genomic DNAs rAAVbiΔBC-EGFP, rAAVwt-EGFP extracted from virions were transfected into BHK21 cells respectively. In contrast with the increased transgene expression caused by infection of rAAVbiΔBC-EGFP, EGFP expression of the cells transfection with either plasmid DNA pAAV2biΔBC-EGFP or genomic DNA rAAVbiΔBC-EGFP extracted from virions was not higher than their respective controls (Fig. 9a,b). The results indicated the expression of rAAVbiΔBC was indistinguishable from rAAVwt when rAAV DNA was introduced either as plasmid DNA or as genomic DNA extracted from virions.

## Discussion

ITR is the  unique *cis* element involved in rAAV packaging. The Rep binding element (RBE), a tandem repeat (GAGC GAGC GAGC GCGC) located in the A-A’ region, plays an important role in ITR function, as it is recognized and bound by Rep78/68^11, 26–28^. Rep has a site-specific DNA helicase activity that unwinds RBE-containing DNA^29^. RBE’, a CTTTG motif at one tip of one of the internal palindromic B-B’ region, can also be bound by Rep78/68^30, 31^. The D sequence at the 3′ end of the AAV genome is the packaging signal^32^.

Musatov *et al*.^33^ found that rAAV harboring only A and D regions can replicate in a circular form and then be packaged as ssAAV. It has been suggested that the B-B’ and C-C’ regions of ITR may not be important for rAAV replication and packaging. To directly assess whether deletion of the B-B’ and C-C’ regions in the two ITRs affects productivity, pAAV2biΔBC was used in rAAV packaging. The results revealed that deletions in the two ITRs did not affect rAAV encapsulation but decreased productivity. In the rAAVbiΔBC genome, even though the B-B’ and C-C’ regions were deleted, the A-A’ region was intact and its ability to be recognized and bound by Rep78/68 was preserved^11^, which may be the primary reason rAAVbiΔBC was still packaged. As a result of the deletion of the B-B’ and C-C’ regions, RBE’ was lost, which may be related to the decrease in productivity. Analogous to RBE, RBE’ is also required for efficient Rep-mediated nicking. Rep makes specific nucleotide contacts with RBE’ that facilitates DNA helicase activity and trs cruciform extrusion during trs nicking^31^. rAAV plasmids with deletions of RBE’ and adjacent sequences have been shown to exhibit lower rates of replication than wt AAV constructs^34^, consistent with the reduced replication of rAAVbiΔBC observed in our study (Fig. 3a,b). Our results suggest that deletion of the B-B’ and C-C’ regions impairs the genome replication of rAAV and ultimately reduces productivity.

Transgene expression of rAAVbiΔBC was higher than rAAVwt both *in vitro* and *in vivo*. Increased expression *in vivo* is a significant advantage, especially in gene therapy. A lower dose of this novel rAAV would provide a better therapeutic response than rAAVwt, which is equivalent to reducing the burden on patients. Compared with traditional rAAVwt, for which infection of some cell lines such as B16F10^35^ is difficult, rAAVbiΔBC also dramatically increased the expression, which further extends the applications of rAAV.

The increased expression of rAAVbiΔBC may be due to differences in transcriptional or post-transcriptional regulation of rAAVbiΔBC and rAAVwt. Because the expression cassettes of the two rAAV vectors were identical, the increased expression was not due to post-transcriptional regulation. Thus, we predicted that the differences in expression may be the result of transcriptional regulation. It has been reported that ATM can affect the expression of rAAV^20^. Subsequent studies have also shown that DNA molecules with one or two HP ends can interact with the ATM-dependent pathway and remain linear and transcriptionally silent, while in the absence of ATM, they undergo circularization and activate expression^21, 22^. Compared with the linear form, the circular rAAV genome is conducive to higher expression^36^. In this study, we determined the effect of ATM on the expression of rAAVbiΔBC. Although the addition of an ATM inhibitor was able to increase the expression of both rAAVwt and rAAVbiΔBC, the rAAVwt increase was significantly higher than rAAVbiΔBC, suggesting that the inhibitory effect of ATM on the expression of rAAV with two wt ITRs was more potent. ATM-dependent inhibition on AAV expression was partially relieved in cells infected rAAV with two ITRΔBC.

Ling *et al*.^37^ showed that deletion of some regions of ITR may affect viral transgene expression through avoiding cellular protein binding. During AAV infection, the MRN complex is recruited to the viral genome shortly after introduction where it dramatically limits virus activity^24^. Previous studies have shown that the MRN complex limits the transduction of AAV by recognizing AAV ITRs through the terminal hairpin structure, and knockdown of proteins of the MRN complex leads to enhancement of the virus^23, 24^. In this study, we showed that inhibition and degradation of the MRN complex contributed to increased expression of rAAVwt and rAAVbiΔBC. However, the increase of rAAVwt was far greater than rAAVbiΔBC, especially when the MRN complex was degraded, suggesting that deletion of the B-B’ and C-C’ regions in the two ITRs reduced MRN-dependent effect of inhibition on AAV expression.

We have proved that the inhibition effect of ATM and MRN on gene expression in U-shaped ITR rAAV would be reduced (Figs 7 and 8a). However, neither the ATM inhibitor nor the Mre11 inhibitor can restore the expression efficiency of rAAVwt to a level of rAAVbiΔBC. There may be more pathways other than ATM or MRN pathway, which contribute to the transgene expression increase of rAAVbiΔBC.

In contrast with the higher expression of rAAVbiΔBC, ITRΔBCs did not increase the expression of rAAV DNA as plasmid or genomic DNA extracted from virions (Figs 4a,b and 9a,b), which can be attributed to the differences in ITR conformation. In genomes within viral particles ITRs form HP conformation, while they form linear double-stranded and open duplex structure, respectively, in rAAV vector plasmid and genomic DNA extracted from virions. DNA sequence of the purified viral genome is identical to that of viral vector carrying genome. If the two of genomes can form the same conformation of ITR, their transgene expression would be similar because some pathways recognize the conformation of ITRs and impose inhibition on rAAV expression^21–24^. However, a purified viral genome is usually annealed and formed double-stranded DNA (dsDNA)^38^. dsDNA can not form the ITR conformation and is similar to plasmid DNA. Thus the gene expression of a purified viral genome is similar to that of rAAV vector plasmid when it is transfected into cells. As Cataldi *et al*.^21^ reported DNA molecules containing wt ITR without HP conformation are not subject to a loss of gene expression. TR HP conformation, but not primary sequence induces ATM-dependent decrease in gene expression, which may be one of the reasons why the expression level of rAAVbiΔBC is similar to rAAVwt introduced into cells either as plasmid or genomic DNA. The results suggest that in addition to the difference of primary structures the specific secondary structure also contribute to the differences in expression between rAAVwt and rAAVbiΔBC as a result of infection.

Due its nonpathogenic nature, the rAAV vector is being used more frequently in gene therapy. However, its genome size limits its application, as the size of inserted foreign DNA is limited to approximately 4.7 kb^39, 40^. The B-B’ and C-C’ regions consisting of 34 nt were deleted from one ITR; therefore, 68 nt could be added to the packaging capacity in rAAVbiΔBC. This could allow for the use of a stronger promoter or an additional enhancer sequence to increase gene expression, or allow for the insertion of larger genes in rAAV vectors.

We found that deletion of the B-B’ and C-C’ regions in the ITRs did not affect the encapsulation of rAAV but reduced its productivity. And the deletion facilitated the expression of rAAV *in vitro* and *in vivo*. As a new truncated ITR, ITRΔBC provides a new option for the design of the rAAV vector genome, although further studies will be necessary to determine the effects of the deletion in ITRs on AAV genome integration. In-depth clarification of the mechanisms underlying the increased expression caused by the truncated ITRs will facilitate the use of rAAVbiΔBC in human gene therapy.

## Methods

### Ethics Statement

The protocol of this study was approved by the Ethics Committee of Jilin University. The animal trials in this study were carried out in accordance with the Regulations for the Administration of Affairs Concerning Experimental Animals approved by the State Council of People’s Republic of China (11-14-1988). All animal procedures were approved by the Institutional Animal Care and Use Committee (IACUC) of Jilin University.

### Cell lines and culture

Murine cell lines, including HEK293, Huh7, B16F10, were obtained from the American Type Culture Collection (Rockville, MD, USA). BHK21 was preserved in our laboratory. All cell lines were maintained as monolayer cultures in Dulbecco’s Modified Eagle’s Medium containing 10% fetal calf serum (FBS), 100 μg/mL penicillin, and 100 U/mL streptomycin, as recommended by the manufacturer (Gibco).

### Construction of the rAAV vector plsmid

The rAAV vector plasmid pAAV2wt harboring two intact wt ITRs was preserved in our laboratory. A truncated ITR lacking the B-B’ and C-C’ regions (ITRΔBC) was synthesized and cloned into pMD18T (Takara, Dalian, China). ITR∆BC was removed from pMD18T to replace both wt ITRs in pAAV2wt to generate pAAV2biΔBC. The ITRΔBC sequence is as follows: tccctctctgcgcgctcgctcgctcactgaggccgccccgcggcctcagtgagcgagcgagcgcgcagagagggagtggccaactccatcactaggggttcct.

### rAAV vector production and purification

rAAV vectors were produced with the Adeno virus-free, triple-plasmid co-transfection method as previously described^13^ with the following modifications. In each batch of rAAV vector production, HEK293 cells were first seeded at 30% confluence in 20 culture dishes with a diameter of 150 mm. The confluence increased to 80% at 2 d post-inoculation. After counting, cells were co-transfected with the rAAV vector plasmid carrying the transgene flanking the ITRs, the AAV RC plasmid harboring the Rep and capsid genes of AAV without the terminal repeats, and the pHelper plasmid harboring the adenovirus helper E2, E4, and VA RNA genes (Ad DNA). Cells were harvested and purified 72 h post-transfection as previously described^14^. Finally, each batch of rAAV vectors was stored in 5 mL of phosphate-buffered saline (PBS).

### rAAV productivity assays

#### SDS-polyacrylamide gel electrophoresis (SDS-PAGE)

rAAV vectors (15 μL) were mixed with 5 μL of 4 × loading buffer, heated in a boiling water bath for 10 min, and resolved by 10% SDS-PAGE.

#### Protein concentration

After rAAV purification, a 10-μL sample was collected to determine the concentration of the capsid protein using the BCA method (Pierce, Rockford, IL, USA).

#### DNA dot blot and qPCR

To accurately measure productivity, the titers of different types of rAAV vectors were detected based on the same number of particles. The concentrations of the capsid protein and the number of particles of the two types of rAAV vectors were adjusted to be identical with the addition of PBS. Titers were then determined with the DNA dot blot method^41^, using a digoxin-labeled CMV promoter and a CAG promoter fragment as the probe. The digoxin-labeled CMV promoter and CAG promoter fragment were synthesized by PCR. The primers used were as follows: CMV-F: 5′-cccataaggtcatgtactgggcat-3′, CMV-R: 5′-gttcccatagtaacgccaataggg-3′, CAG-F: sequence identical to CMV-F, and CAG-R: sequence identical to CMV-R. The viral titers were also assayed by qPCR as previously described^42^. The same primers were used for qPCR as for the DNA dot blot assays.

#### Southern blotting of the rAAV vector genome

Genomic DNA of the rAAV vector was extracted, purified, dissolved in 100 mM NaOH and 1 mM EDTA for denaturation, and loaded onto an alkaline denaturing agarose gel (1%) for electrophoresis. Southern hybridization was performed using a GeneScreen Plus membrane, according to a standard protocol (Molecular Cloning, 3^rd^ edition). The probe used was a digoxin-labeled CMV promoter fragment.

### Replication efficiency assays of the rAAV genome

HEK293 cells were first seeded at 30% confluence in a culture dish with a diameter of 100 mm. The confluence was increased to 80% at 2 d post-inoculation. After counting, cells were co-transfected with pcDNA3.1-p5-Rep, pHelper, and pAAV2wt-EGFP/pAAV2biΔBC-EGFP plasmids. Hirt DNA was extracted and resuspended in 100 μL of TE buffer at 12, 24, 36, 48, and 72 h post-transfection, and 2% of the total yield was digested with *Dpn*I in a 20-μL reaction overnight. The genomic copy number of the samples was assayed by qPCR, with the following primers: CMV-EGFP-F: 5′-ctcgtttagtgaaccgtcag-3′ and CMV-EGFP-R: 5′-aacagctcctcgcccttg-3′.

In another comparison of the replication efficiency of the rAAV genome, HEK293 cells were seeded as described above and co-transfected with pcDNA3.1-p5-Rep and pHelper plasmids. The cells were infected by rAAVDJwt-EGFP and rAAVDJΔBC-EGFP at an MOI of 200 vg/cell at 12 h post-transfection, respectively. Hirt DNA was extracted and resuspended in 100 μL of TE buffer at 3, 12, 24, 36, 48, and 72 h post-infection. The genomic copy number of the samples was assayed by qPCR.

#### Hirt DNA extraction

Viral DNA was isolated from cells with the Hirt extraction method as previously described^24^, with the following modifications. Cells were suspended in 765 μL of Hirt solution (10 mM Tris-HCl pH 7.4, 100 mM EDTA), 1% SDS and 5 M NaCl were added to a total volume of 850 μL, and the sample was mixed and incubated at 4 °C for 24 h. Precipitated material was pelleted by centrifugation at 16,000 × *g* for 30 min at 4 °C, and the supernatant was extracted using an equal volume of phenol-chloroform (1:1). DNA was precipitated from the aqueous layer with the addition of one volume of isopropyl alcohol and 0.2 volumes of 5 M potassium acetate at 4 °C for 30 min. After centrifugation at 16,000 × *g* for 5 min, DNA was resuspended in 100 μL of TE buffer.

### rAAV expression assays

#### *In vitro* expression

For *in vitro* assays, 1.0 × 10^6^ HEK293 cells were seeded in each well of a six-well plate 12 h prior to infection. rAAV-EGFP was added in a series of MOIs ranging from 100 to 10,000 vg/cell. EGFP expression was detected 48 h post-infection with a BD FACSCalibur Flow Cytometer. The level of EGFP expression was analyzed by FlowJo software v. 7.6.2. In other assays, BHK21, Huh7, and B16F10 cells were infected with rAAV-EGFP at an MOI of 1,000 vg/cell. EGFP expression was detected at 24, 48, and 72 h post-infection. Detection of β-galactosidase activity was performed according to a standard protocol (Molecular Cloning, 3^rd^ edition).

#### *In vivo* expression

For *in vivo* assays, four-week-old male C57BL/6 J mice were used (HFK Bioscience Co., Beijing, China). To assay Gluc activity, 200 μL (2.0 × 10^11^ vg, 2.0 × 10^10^ vg, and 2.0 × 10^9^ vg) of either rAAV8wt-Gluc or rAAV8biΔBC-Gluc was injected into the mice through the tail vein. Gluc activity was measured with the Gaussia Luciferase Assay kit (New England Biolabs) according to the manufacturer’s instructions. Briefly, 50 μL of substrate solution was added to 2.5 μL of tail-vein blood and relative luciferase units (RLU) were determined using a luminometer.

#### In vitro expression in the presence of ATM or Mre11 inhibitor

To detect the effect of ATM on rAAV expression, BHK21 cells were seeded at 30% confluence in each well of a six-well plate and treated with 10 μM ATM inhibitor (KU55933; Selleck, USA) or 10 μM dimethyl sulfoxide (DMSO) on day 1, and the cells were infected with rAAV8wt-Gluc and rAAV8biΔBC-Gluc on day 2, respectively. Gluc activity was measured 48 h post-infection with the Gaussia Luciferase Assay kit (New England Biolabs) according to the manufacturer’s instructions. Briefly, 50 μL of substrate solution was added to 20 μL of culture medium and RLU were determined using a luminometer. Cells were maintained in the presence of the ATM inhibitor or DMSO until Gluc activity was assayed. To detect the effect of the MRN complex on rAAV expression, BHK21 cells were seeded and infected with rAAV as described above, but treated with 25 μM Mre11 inhibitor (Mirin, Sigma, USA) or 25 μM DMSO.

The effect of MRN degradation on AAV expression was also evaluated. HEK293 cells were seeded at 30% confluence in each well of a six-well plate, and transfected with pHelper plasmids 48 h later. The cells were infected with rAAV8wt-Gluc and rAAV8biΔBC-Gluc after counting, 12 h post-transfection. Finally, 48 h post-transfection, Gluc activity was measured as described above.

### Statistical analysis

All results are expressed as means ± standard error (SEM) calculated over a minimum of three or more independent experiments. Significant differences were estimated using Student’s t tests and are noted as * and ** for *p-values* < 0.05 and <0.01, respectively.

## Electronic supplementary material

Supplementary Information
