# Supplementary material for: Deletion of the B-B’ and C-C’ regions of inverted terminal repeats reduces rAAV productivity but increases transgene expression
Source: Sci Rep. 2017 Jul 14;7:5432. doi: 10.1038/s41598-017-04054-4 (PMC5511163; doi:10.1038/s41598-017-04054-4)
Supplement: Supplementary file 1 — Supplementary Information [file 41598_2017_4054_MOESM1_ESM.pdf]

Supplementary Information

Deletion of the B-B' and C-C' regions of inverted terminal repeats reduces rAAV productivity but increases transgene expression

Qingzhang Zhou[1], Wenhong Tian[2], Chunguo Liu[3, 4], Zhonghui Lian[1], Xiaoyan Dong[4] & Xiaobing Wu[1, 2]

[1]College of Life Sciences, Jilin University, Changchun, Jilin, China. [2]Beijing Ruicy Gene Therapy Institute for Rare Diseases, Beijing, China. [3]College of Life Science and Technology, Beijing University of Chemical Technology, Beijing, China. [4]Beijing FivePlus Molecular Medicine Institute, Beijing, China. Correspondence and requests for materials should be addressed to X.W. (email: wuxb0168@vip.sina.com).

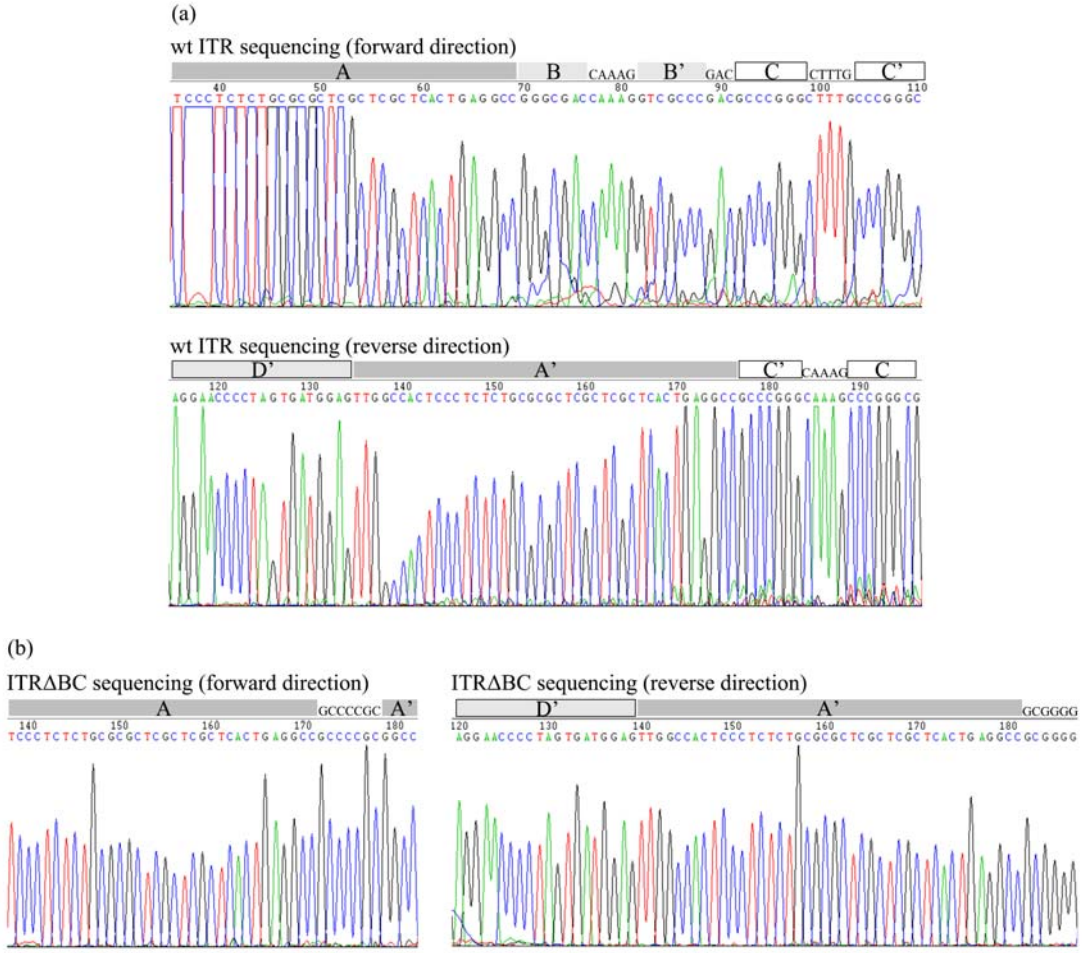

Figure S1. Sequences of wt ITR and truncated ITR lacking the B-B' and C-C' regions

were confirmed by bidirectional DNA sequencing. (a) Sequencing results of wt ITR in pAAV2wt. Results of sequencing of the forward direction (up) and the reverse direction (down) were overlapped by 20 base pairs (bp). (b) Sequencing results of ITRΔBC in pAAV2biΔBC. Results of sequencing of the forward direction (left) and the reverse direction (right) were overlapped by 10 bp. Only one ITR in the upstream direction of the expression cassette is shown. The second ITR was identical.

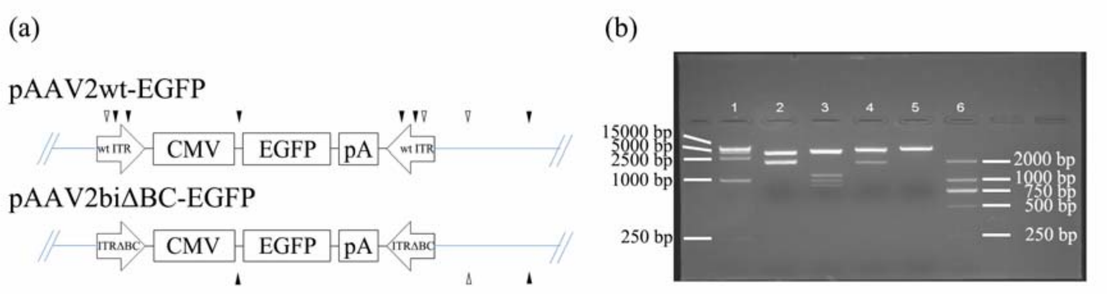

Figure S2. rAAV vector plasmids were confirmed by restriction enzyme digestion. (a) Restriction maps of the rAAV vectors. In pAAV2wt-EGFP and pAAV2biΔBC-EGFP, *Ahd*I and *Sma*I restriction sites are indicated by hollow and solid arrows, respectively. DNA fragments of the predicted sizes from each of two plasmids are indicated. Following cleavage with *Ahd*I, pAAV2wt-EGFP generated three bands of 2.0, 1.8, and 3.9 kilobases (kb) while pAAV2biΔBC-EGFP generated only one band of 7.7 kb. Following cleavage with *Sma*I, pAAV2wt-EGFP generated four large bands of 0.8, 1.1, 4.8, and 1.0 kb and two small bands of 11 bp while pAAV2biΔBC-EGFP generated only two bands of 5.8 and 1.9 kb. (b) Electrophoretic analysis of the structural integrity of wt ITRs and ITRΔBCs in rAAV vector plasmids. Following digestion with *Ahd*I and *Sma*I, the DNA fragments were analyzed by 2% neutral agarose gel electrophoresis. Lane 1, DNA marker DL15000 (Takara, Dalian, China); lane 2, pAAV2wt-EGFP digested with *Ahd*I; lane 3, pAAV2wt-EGFP digested with *Sma*I; lane 4, pAAV2biΔBC-EGFP digested with *Sma*I; lane 5, pAAV2biΔBC-EGFP digested with *Ahd*I; and lane 6, DNA marker DL2000 (Takara, Dalian, China).

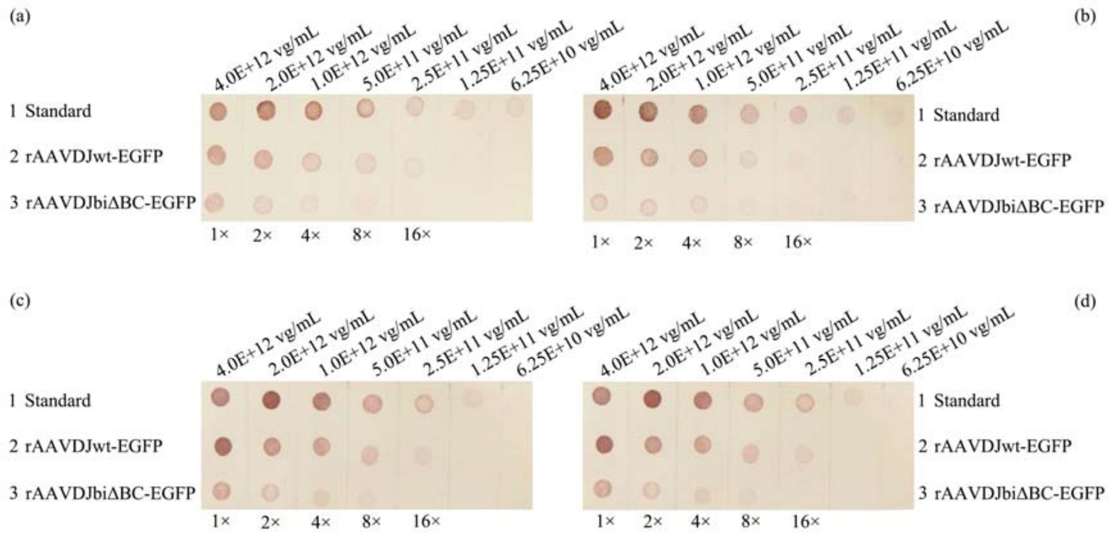

Figure S3. Titers of rAAVDJwt-EGFP and rAAVDJbiΔBC-EGFP. DNA dot blot assay of the second (a), third (b), fourth (c) and fifth (d) batches of rAAVDJwt-EGFP and rAAVDJbiΔBC-EGFP. The result of first batch was shown in Fig. 2c.

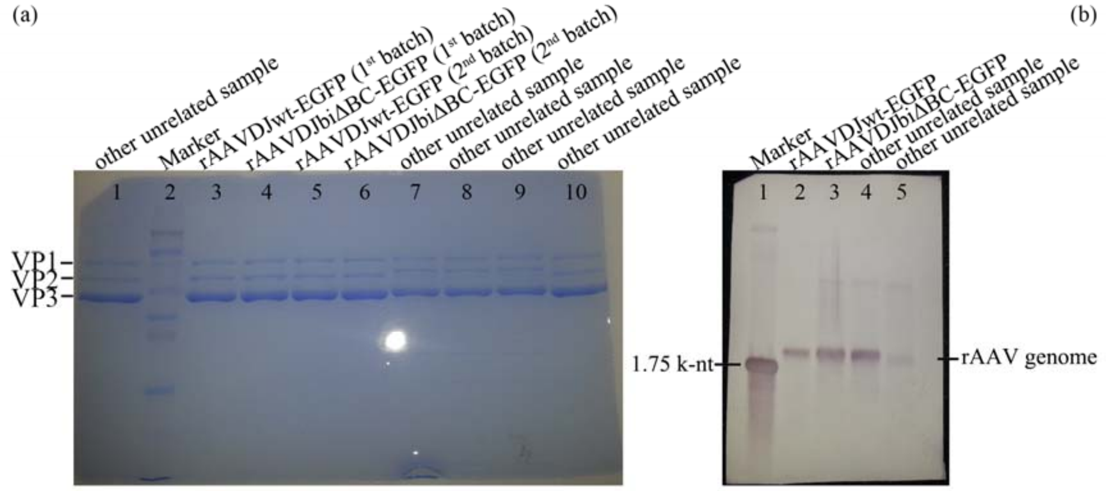

Figure S4. Original full-length pictures of SDS-PAGE and Southern blotting. (a) SDS-PAGE analysis of the capsid of the rAAVDJwt-EGFP and rAAVDJbiΔBC-EGFP. Lane 2, Protein marker, PageRuler Prestained Protein Ladder, 10 - 180 kDa (Thermo Scientific, China); lane 3 and 5, the first and second batches of rAAVDJwt-EGFP; lane 4 and 6, the first and second batches of rAAVDJbiΔBC-EGFP; lane 1, 7, 8, 9 and 10, other unrelated samples. (b) Genomic integrity of rAAVDJwt-EGFP and rAAVDJbiΔBC-EGFP was determined by Southern blotting. Lane 1, marker; lane2 and 3, the first batch of rAAVDJwt-EGFP and rAAVDJbiΔBC-EGFP; lane 4 and 5,

other unrelated samples.

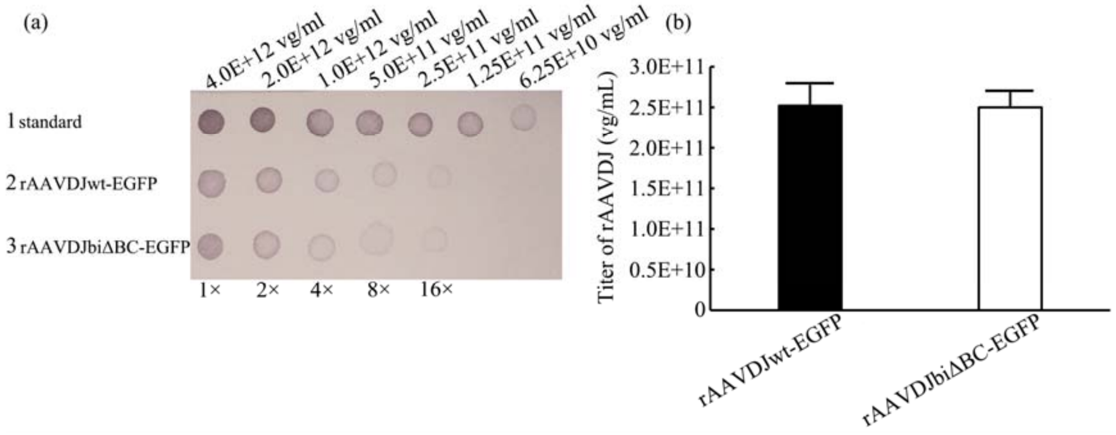

Figure S5. Adjustment of the rAAV titers. After being adjusted to be identical, the titers of rAAVDJwt-EGFP and rAAVDJbiΔBC-EGFP were detected by DNA dot blot assay (a) and qPCR (b).

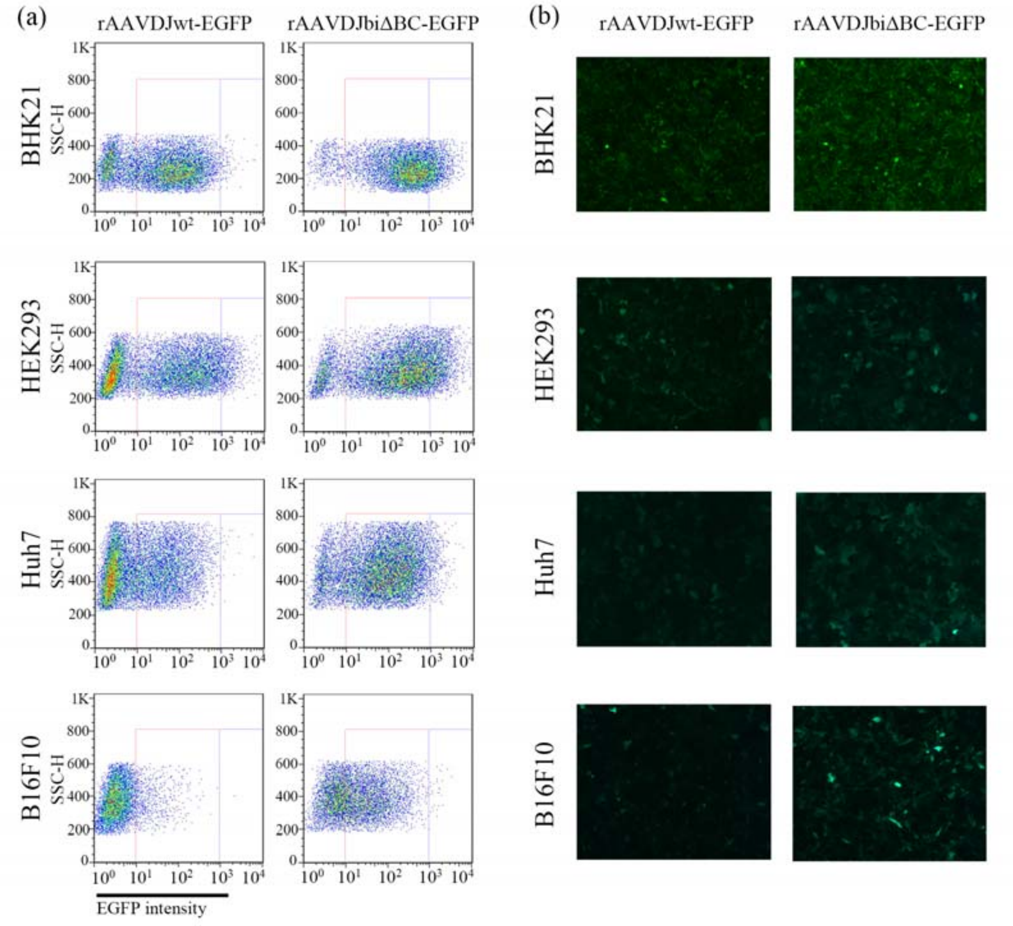

Figure S6. Superior *in vitro* expression of rAAVbiΔBC. (a) Flow cytometry

scatterplot of positive BHK21, HEK293, Huh7, and B16F10 cells infected with 1,000 vg/cell rAAVDJwt-EGFP or rAAVDJbiΔBC-EGFP for 48 h. (b) Micrographs of BHK21, HEK293, Huh7, and B16F10 cells infected with rAAVDJwt-EGFP or rAAVDJbiΔBC-EGFP at an MOI of 1,000 vg/cell for 48 h, respectively.

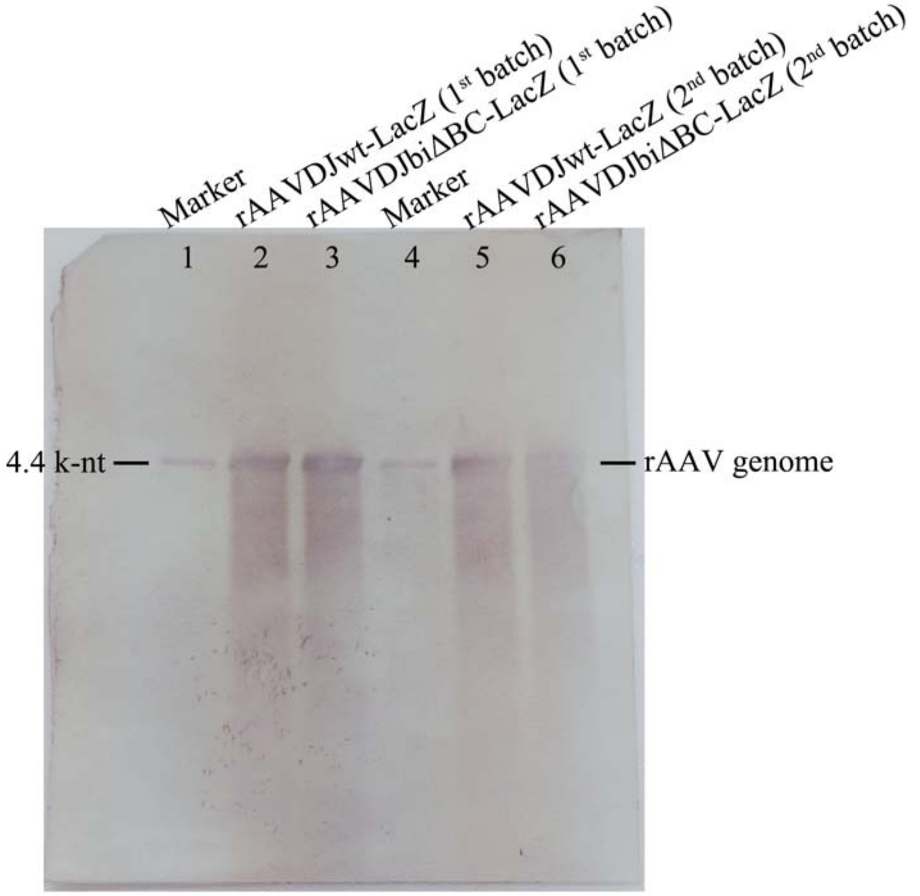

Figure S7. Original full-length picture of genomic integrity of rAAVDJwt-LacZ and rAAVDJbiΔBC-LacZ. Genomic integrity of rAAVDJwt-LacZ and rAAVDJbiΔBC-LacZ was determined by Southern blotting. Lane 1 and 4, marker; lane2 and 5, the first and second batches of rAAVDJwt-LacZ; lane 3 and 6, the first and second batches of rAAVDJbiΔBC-LacZ.

Table S1. Titers of five batches of rAAVDJwt-EGFP and rAAVDJbiΔBC-EGFP (vg/mL)

	No.1	No.2	No.3	No.4	No.5
rAAVDJwt-EGFP	1.01×10^{12}	9.10×10^{11}	9.32×10^{11}	1.18×10^{12}	1.23×10^{12}
rAAVDJbiΔBC-EGFP	2.63×10^{11}	2.21×10^{11}	2.29×10^{11}	2.65×10^{11}	2.70×10^{11}
